# Effects of three types of fresh *Rehmannia glutinosa* improve lipopolysaccharide-induced acute kidney injury in sepsis through the estrogen receptor pathway

**DOI:** 10.22038/IJBMS.2023.67322.14757

**Published:** 2023

**Authors:** Meng Liu, Mengnan Zeng, Pengli Guo, Yuhan Zhang, Xiaofeng Yang, Jufang Jia, Qinqin Zhang, Beibei Zhang, Bing Cao, Ru Wang, Xiaoke Zheng, Weisheng Feng

**Affiliations:** 1 Department of Medicine, Henan University of Chinese Medicine, Zhengzhou 450046, China; 2 The Engineering and Technology Center for Chinese Medicine Development of Henan Province, Zhengzhou 450046, China

**Keywords:** Acute kidney injury, Estrogen, Rehmannia glutinosa, Lipopolysaccharide, NF-kappa B, NLRP3, Sepsis, TLR4

## Abstract

**Objective(s):**

To explore the effects and mechanism of three types of fresh *Rehmannia glutinosa*, namely Beijing No. 3 (BJ3H), Huaizhong No. 1 (HZ1H), and Taisheng (TS) on lipopolysaccharide (LPS)-induced acute kidney injury in the sepsis (S-AKI) mice model through the estrogen receptor pathway.

**Materials and Methods:**

BALB/c mice were randomly divided into control (CON), model (LPS), astragalus injection (ASI), BJ3H, HZ1H, TS water extract groups, the estrogen receptor antagonist ICI182,780 groups were added to each group. The antagonist groups received an intraperitoneal injection of ICI 0.5 hr before administration and an intraperitoneal injection of LPS 3 days after administration. The kidney pathology, function, inflammatory factors, immune cells, levels of reactive oxygen species (ROS), apoptosis, and the protein expression levels of TLR4/NF-κB/NLRP3 signaling pathway in the mice kidneys were detected.

**Results:**

ASI, BJ3H, HZ1H, and TS improved LPS-induced renal pathology in S-AKI mice, reduced the kidney and serum levels of inflammatory factors, positive rates of macrophages and neutrophils, levels of ROS and apoptosis, and the relative expression levels of TLR4, MyD88, NF-κB p-p65/NF-κB p65, and NLRP3 proteins in the kidney. In addition, they increased the positive rate of dendritic cells (DCs) in the mice kidneys. The overall effect of HZ1H was superior to that of ASI, BJ3H, and TS. However, after adding ICI, the regulatory effects of drugs were inhibited.

**Conclusion:**

The three types of fresh *R. glutinosa* may completely or partially affect the TLR4/NF-κB/NLRP3 signaling pathway through the estrogen receptor pathway to exert a protective effect on S-AKI.

## Introduction

Sepsis is a life-threatening disorder with a complex pathogenesis induced by an extreme and systemic inflammatory response to an infection (1). According to the 2018 World Health Organization (WHO) report, more than 30 million people worldwide develop sepsis every year (2). Sepsis is considered the main cause of acute kidney injury (AKI) in severely ill patients. A previous study reported that about 40‒50% of patients with sepsis develop sepsis-induced AKI (S-AKI), which is associated with a mortality rate of 30‒60% in intensive care unit patients (2, 3). However, so far, no effective treatment has been developed to significantly reduce the mortality from S-AKI. Therefore, exploring mechanisms involved in S-AKI to identify new therapeutic drugs is necessary.

In recent years, a large number of studies have been conducted on traditional Chinese medicine (TCM). For example, it has been found that TCM drugs with estrogen-like activity, such as yam, may exert a protective effect against S-AKI through the estrogen receptors (4), indicating that estrogen may protect against S-AKI. Estrogen is a hormone with a wide range of biological activities in humans and animals, while phytoestrogens are a class of substances derived from plants with fewer side effects, better safety profile, and higher effectiveness that exert estrogen-like activity by binding to the estrogen receptors (5). *Rehmannia glutinosa* Libosch. is the fresh tuberous root of the Scrophulariaceae plant, which is one of the four major Huai herbal medicines, and acts on the heart, liver, and kidneys (6). It is used in the treatment of diseases classified as “high fever” in TCM and may have a therapeutic effect in S-AKI. In addition, our previous research found that the water extract of fresh *R. glutinosa* has estrogen-like activity (7). Moreover, the research team at Henan University of Chinese Medicine used systematic screening to select the excellent variant individual plants from the population of *R. glutinosa* Beijing No. 3 (BJ3H), and cultivated the new varieties Huaizhong No. 1 (HZ1H; variety identification number: Henan Chinese Medicines 2018001) and Taisheng (TS; variety identification number: Henan Chinese Medicines 2019013). At present, *R. glutinosa* BJ3H is the main variety of Huai *R. glutinosa* and is the most widely circulated in the market. HZ1H and TS have a better appearance than *R. glutinosa* BJ3H, which may have better medicinal effects. Our research group also systematically separated and identified the chemical components of fresh *R. glutinosa* HZ1H and TS, and found that their chemical components, such as amides, terpenes, and ionones, have good anti-inflammatory activity in kidney cells (8-11). However, the specific effects and comparisons among the three types of fresh *R. glutinosa* on S-AKI have not yet been reported. Therefore, this study explored the intervention effects and the underlying mechanisms of the three types of fresh *R. glutinosa*, namely BJ3H, HZ1H, and TS, on the lipopolysaccharide (LPS)-induced S-AKI mice model through the estrogen receptor pathway, which may provide an experimental basis for the clinical treatment of S-AKI.

## Materials and Methods

***Animals***

BALB/c mice (female, aged 6 weeks, 16‒18 g, n=96) were purchased from Beijing Vitalstar Biotechnology Co., Ltd. (protocol number: SCXK [Jing] 2021-0006) and housed in a clean animal laboratory at 18‒22 °C under a 12-hr light/dark cycle with free access to food and water supply. The animal experiments, approved by the Ethical Committee of the Henan University of Chinese Medicine, China (approval no.: DWLL2018080003), were performed in line with the guidelines of the Chinese Ethics Committee.

***Drugs and reagents***

The medicinal materials, fresh *R. glutinosa* BJ3H, HZ1H (variety identification no.: Henan Zhongyaojian 2018001) and TS (variety identification no.: Henan Zhongyaojian 2019013) were harvested from Wenxian County (Henan, China), Wuzhi County (Henan, China), and Wenxian County (Henan, China), respectively. They were identified by Prof. Chengming Dong of Henan University of Chinese Medicine as the fresh and dried roots of *R. glutinosa* Libosch.

***Preparation of medicinal extracts***

Preparation of BJ3H, HZ1H, and TS fresh *R. glutinosa* water extracts: 300 g of fresh *R. glutinosa* medicinal material was weighed and dissolved in water. Then, the juice was squeezed and filtered to obtain a filtrate, concentrated under reduced pressure, and dried. The fresh *R. glutinosa* extraction rates were 13.58%, 15.49%, and 12.14% for BJ3H, HZ1H, and TS, respectively.

***S-AKI mice model induced by LPS and the drugs administered***

BALB/c mice (females, n = 96) were adaptively fed for 1 week and randomly divided into control (CON), model (LPS, L2880, Sigma-Aldrich, St. Louis, MO, USA), astragalus injection (ASI, astragalus injection/original medicinal materials, 4 g/kg, Z23020862, Harbin Zhenbao Pharmaceutical Co., Ltd., Heilongjiang, China; each 20 ml was equivalent to 40 g of the original medicinal material), BJ3H fresh *R. glutinosa* water extract (10 g/kg), HZ1H fresh *R. glutinosa* water extract (10 g/kg), and TS fresh *R. glutinosa* water extract (10 g/kg) groups, and each group added antagonist ICI182,780 (ICI, 0.5 mg/kg, Lot: 67873, MCE Inhibitor Company, Shanghai, China) groups according to the principle of body weight balance, a total of 12 groups with 8 mice in each group. The antagonist groups received an intraperitoneal injection of ICI 0.5 hr before daily administration. Three days after the intragastric administration, LPS was dissolved in normal saline and injected intraperitoneally at a dose of 5 mg/kg to establish the S-AKI mice model. The mice in the CON group were injected with equal doses of normal saline. The intragastric dose of fresh *R. glutinosa* is the weight of the crude drug, and the dosage was calculated as 20-fold higher than the human dosage prescribed in the 2020 edition of the Chinese Pharmacopoeia (6). Six hours after induction of S-AKI in mice, they were anesthetized with isoflurane, their blood was collected, and they were sacrificed by cervical dislocation. The serum and kidneys were collected for subsequent index detection.

***Renal pathology and function-related indicators***

The left kidney was obtained, immersed in 3.7% paraformaldehyde solution for fixation, dehydrated in ethanol, embedded in paraffin, sliced at a thickness of 5 μm, and stained with hematoxylin and eosin (H&E) to detect the renal histopathological changes. The serum of mice was collected and centrifuged at 3000 r/min for 10 min, and the supernatant was collected. The serum levels of creatinine (Cr, C011-2-1, Nanjing Jiancheng Bioengineering Institute, Nanjing, China), urea nitrogen (BUN, C013-2-1, Nanjing Jiancheng Bioengineering Institute, Nanjing, China), and Mouse kidney injury molecule 1 (Kim-1, MM-0318M1, Jiangsu Enzyme Immunoassay Industry Co., Ltd., Nanjing, China) in the mice were detected using the kits, according to the manufacturer’s instructions.

***Serum levels of IL-6, TNF-α, IL-1β, and MCP-1 in mice***

The mice serum levels of inflammatory factors, including interleukin 6 (IL-6, MM-0163M1, Jiangsu Enzyme Immunoassay Industry Co., Ltd., Nanjing, China), tumor necrosis factor-alpha (TNF-α, MM-0132M1, Jiangsu Enzyme Immunoassay Industry Co., Ltd., Nanjing, China), interleukin 1β (IL-1β, MM-0040M1, Jiangsu Enzyme Immunoassay Industry Co., Ltd., Nanjing, China) and monocyte chemoattractant protein 1 (MCP-1/CCL2/MCAF, MM-0082M1, Jiangsu Enzyme Immunoassay Industry Co., Ltd., Nanjing, China) were detected using ELISA kits in strict accordance with the manufacturers’ instructions.

***Positive rate of dendritic cells (DCs) in the primary kidneys of mice***

The right kidney was freshly cut into slices after quickly removing the kidney capsule. Phosphate-buffered saline (PBS) was added to wash the samples and the supernatant was collected by centrifugation; this process was repeated twice. Then, 1 ml of trypsin was added to digest the sample, and fetal bovine serum was added after 3 min to terminate the digestion. The filtrate was collected by filtering through a 70-μm filter and centrifuged at 1500 r/min for 5 min. The supernatant was discarded to obtain the primary kidney cells, and 100 μl of cells were resuspended in PBS and transferred to the flow tube. Antibodies against the surface markers CD11c (2213759, Thermo Fisher Scientific, Shanghai, China) and CD86 (2213051, Thermo Fisher Scientific, Shanghai, China) of DCs were added. After being vortexed, the cells were incubated at room temperature for 30 min in the dark, and 2 ml of PBS was added. Then, the sample was centrifuged at 300×g for 5 min, and the supernatant was discarded. This process was repeated twice. At last, 300 µl of PBS was added after the final centrifugation and flow cytometry (BD Biosciences, San Jose, CA, USA) was performed.

***Immunohistochemical staining to detect the positive rate of IL-6, TNF-*** α***, macrophages, and neutrophils in the mice kidneys***

The mice’s kidney sections were baked at 60 °C in an oven, dewaxed, and rehydrated. Then, the antigens were repaired by high temperature and high pressure. Peroxidase was blocked at room temperature for 25 min, and serum was blocked for 30 min. The primary antibodies IL-6 (1:100, A0826, Abclonal, Wuhan, China), TNF-α (1:100, A11534, Abclonal, Wuhan, China), F4/80 (1:800, GB11027, Servicebio, Wuhan, China), and Ly-6G (1:600, GB11229, Servicebio, Wuhan, China) were added dropwise to the sections and incubated overnight at 4 °C. Then, the secondary antibody (GB23303, Servicebio, Wuhan, China) was added and incubated at room temperature for 50 min. After DAB staining, the cell nuclei were counterstained with hematoxylin, dehydrated, and mounted. Microscopic examination (XSP-C204, CIC, Weztlar, Germany) and image acquisition as well as analysis were performed. Image-Pro Plus 6.0 was used to analyze the images, in which the hematoxylin-stained nuclei were stained blue, whereas DAB expression appeared brown.

***Flow cytometry analysis of ROS and apoptosis in the primary kidney cells of mice***

The primary kidney cells were divided into two parts. One part was prepared according to the instructions of the ROS detection kit (20191220, Solarbio, Beijing, China). The change in the cellular ROS level was detected and analyzed by flow cytometry (BD Biosciences, San Jose, CA, USA). The other part was transferred to the flow tubes and centrifuged, and the supernatant was discarded. Then, 100 μl of 1×Binding Buffer was added to each tube. The apoptosis rate was detected and analyzed by the AnnexinV-PE/7-AAD method (0020694, Solarbio, Beijing, China) and detected by flow cytometry (BD Biosciences, San Jose, CA, USA).

***Detection of TLR4/NF-κB/NLRP3 signaling pathway-related protein expression by Western blotting***

The kidney tissues were collected and the total protein was extracted from the kidneys of mice in each group using the total protein extraction kit (20211209, Solarbio, Beijing, China). The total protein concentration was determined using the BCA protein quantitative kit (20211213, Solarbio, Beijing, China). PAGE electrophoresis was performed to separate the proteins into different bands according to the relative molecular mass, transferred to the PVDF membrane, and blocked with 5% BSA blocking solution on a shaker for 1 hr. Then, we added the antibodies against TLR4 (1:2000, ab22048, Abcam, Cambridge, UK), MyD88 (1:500, ab135693, Abcam, Cambridge, UK), NF-κB p-p65 (1:5000, ab86299, Abcam, Cambridge, UK), NF-κB p65 (1:2000, ab16502, Abcam, Cambridge, UK), NLRP3 (1:1000, ab4207, Abcam, Cambridge, UK), and β-actin (1:50000, ab8226, Abcam, Cambridge, UK). The mixture was incubated at room temperature for 0.5 hr and at 4 °C overnight in the refrigerator. Then, we added goat anti-rabbit secondary antibody (1:20000, 926-32211, LI-COR, USA) or goat anti-mouse secondary antibody (1:20000, 926-32210, LI-COR, USA), and incubated the solution at room temperature for 1 hr in the dark. The protein bands were scanned and analyzed using the Odyssey dual-color infrared laser imaging system (Li-COR Biosciences Co, UK).

***Data analysis***

SPSS 26.0 statistical software was used for data processing. The data are expressed as mean ± standard deviation (± *s*). One-way analysis of variance (one-way ANOVA) and LSD-t multiple tests were used to determine the group differences. *P*<0.05 was considered statistically significant.

## Results

***Three types of fresh Rehmannia glutinosa improved the pathological and functional indicators in the LPS-induced S-AKI mice***

**Figure 1:**
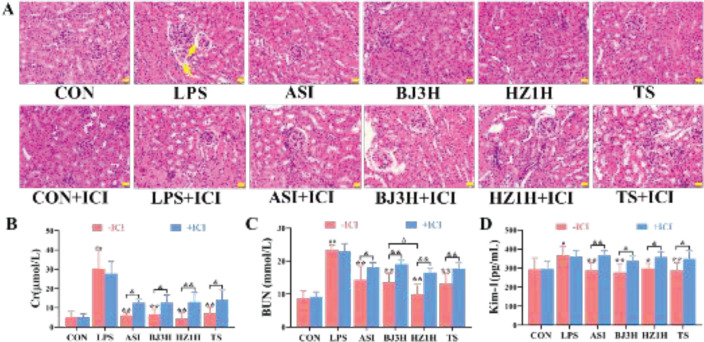
Three types of fresh *Rehmannia glutinosa* improved the pathological and functional indicators in LPS-induced S-AKI mice. (A) H&E staining of the mice kidneys (400×). (B‒D) Serum levels of Cr, BUN, and Kim-1 in the mice. n = 6. CON: normal control group; LPS: model group; ASI: astragalus injection group; BJ3H: Beijing No. 3 fresh R. glutinosa water extract group; HZ1H: Huaizhong No. 1 fresh *R. glutinosa* water extract group; TS: Taisheng fresh *R. glutinosa* water extract group. ICI: ICI182,780. #*P*<0.05, ##*P*<0.01 compared with the CON (-ICI) group. *P<0.05, **P<0.01 compared with the LPS (-ICI) group. Δ*P*<0.05 compared with the BJ3H (-ICI) group. &*P*<0.05, &&*P*<0.01 for comparison between the groups (± ICI). Scale bar = 20 µm

The results of the H&E staining showed that there were no abnormal pathological changes in the kidneys of the CON group mice. Glomerular atrophy, tubular dilatation, and inflammatory cell infiltration were observed in the LPS-induced mice kidneys, as indicated by yellow arrows (Figure 1A). ASI, BJ3H, HZ1H, and TS significantly reduced LPS-induced kidney injury. However, these effects were completely or partially blocked by ICI, while there were no effects on the CON mice kidneys. In addition, the serum levels of Cr, BUN, and Kim-1 were significantly increased (Figure 1B‒D,* P*<0.05, *P*<0.01) in the model group compared with the control group. The levels of Cr, BUN, and Kim-1 were significantly decreased in each administration group (*P*<0.05, *P*<0.01) compared with the LPS group. Moreover, HZ1H had a better effect on reducing the BUN level than BJ3H and TS (*P*<0.05). The effects in the treatment groups were completely or partially inhibited by ICI (*P*<0.05, *P*<0.01), which indicated that the three types of *R. glutinosa* may completely or partially improve the renal pathology and function damage in LPS-induced S-AKI mice through the estrogen receptor pathway.

***Three types of fresh R. glutinosa alleviated the levels of inflammatory factors in LPS-induced S-AKI mice***

**Figure 2:**
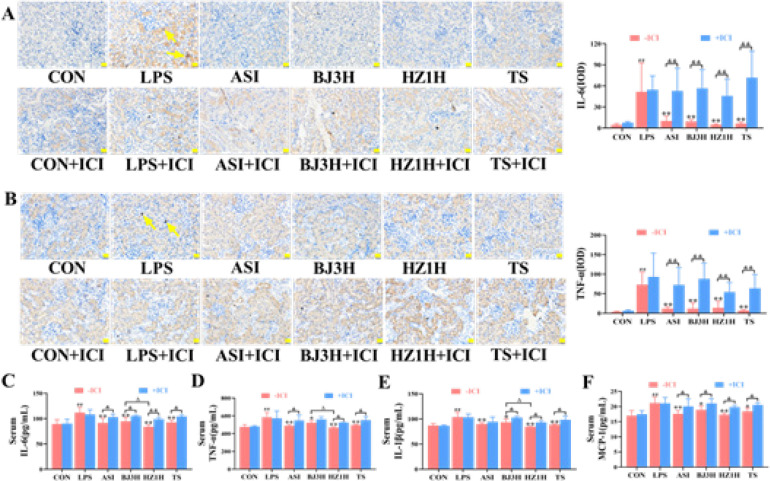
Three types of fresh *Rehmannia glutinosa* alleviated the levels of inflammatory factors in the LPS-induced S-AKI mice. (A‒B) The positive rates of IL-6 and TNF-α in the mice kidneys, respectively. The yellow arrows in panels A and B indicate positive expression of IL-6 and TNF-α, respectively. n = 8. (C‒F) Levels of IL-6, TNF-α, IL-1β, and MCP-1 in mice serum. n = 6. CON: normal control group; LPS: model group; ASI: astragalus injection group; BJ3H: Beijing No. 3 fresh *R. glutinosa* water extract group; HZ1H: Huaizhong No. 1 fresh *R. glutinosa* water extract group; TS: Taisheng fresh *R. glutinosa* water extract group. ICI: ICI182,780. ##*P*<0.01 compared with the CON (-ICI) group. *P<0.05, **P<0.01 compared with the LPS (-ICI) group. Δ*P*<0.05 compared with the BJ3H (-ICI) group. &*P*<0.05, &&*P*<0.01 for comparison between the groups (± ICI). Scale bar = 20 µm

LPS can trigger the systemic inflammatory response, and inflammation is a hallmark of S-AKI. The results of immunohistochemical staining suggested that ASI, BJ3H, HZ1H, and TS reduced the positive rates of IL-6 and TNF-α in the mice kidneys induced by LPS (Figure 2A‒B, *P*<0.01). In addition, the ELISA method was performed to detect the inflammatory factors in the mice serum, which showed that the serum levels in the model group of pro-inflammatory cytokines IL-6, TNF-α, IL-1β, and MCP-1 were significantly increased (Figure 2C‒F, *P*<0.01) in comparison with the control group. ASI, BJ3H, HZ1H, and TS significantly alleviated the serum levels of pro-inflammatory cytokines in mice (*P*<0.05, *P*<0.01) compared with the LPS group. Moreover, the overall effects of HZ1H were superior to those of BJ3H and TS (*P*<0.05). The regulatory effects of each administration group were completely or partially inhibited by adding ICI (*P*<0.05, *P*<0.01), suggesting that the three types of fresh *R. glutinosa* may completely or partially reduce the levels of inflammation in LPS-induced S-AKI mice through the estrogen receptor pathway.

***Three types of fresh R. glutinosa regulated the kidney immune cells in LPS-induced S-AKI mice***

**Figure 3:**
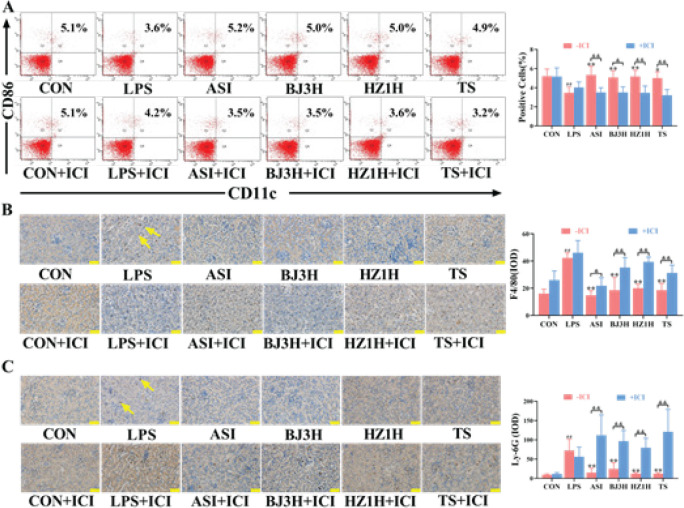
Three types of fresh *Rehmannia glutinosa *regulated the kidney immune cells in LPS-induced S-AKI mice. (A) The pictures and quantitative results of the positive rate of DCs in the mice kidneys. n = 3. (B‒C) The positive rates of macrophages and neutrophils in the mice kidneys, respectively. The yellow arrows in panels B and C indicate positive expression of macrophages and neutrophils, respectively. n = 8. CON: normal control group; LPS: model group; ASI: astragalus injection group; BJ3H: Beijing No. 3 fresh *R. glutinosa *water extract group; HZ1H: Huaizhong No. 1 fresh *R. glutinosa* water extract group; TS: Taisheng fresh *R. glutinosa* water extract group. ICI: ICI182,780. ##*P*<0.01 compared with the CON (-ICI) group. *P<0.05, **P<0.01 compared with the LPS (-ICI) group. &*P*<0.05, &&*P*<0.01 for comparison between the groups (± ICI). Scale bar = 50 µm

The results of flow cytometry and immunohistochemical staining showed that ASI, BJ3H, HZ1H, and TS increased the positive rate of DCs in the primary kidney cells (Figure 3A, *P*<0.05, *P*<0.01), decreased the positive rates of macrophages and neutrophils in the kidneys (Figure 3B‒C, *P*<0.01) induced by LPS. The three types of fresh *R. glutinosa* water extracts had no significant difference in terms of regulating the levels of immune cells. However, the regulating effects of each administration group were completely or partially blocked after adding ICI (Figure 3A‒C, *P*<0.05, *P*<0.01). The results demonstrated that the three types of *R. glutinosa* may completely or partially regulate the levels of renal immune cells in LPS-induced S-AKI mice through the estrogen receptor pathway.

***Three types of fresh R. glutinosa reduced the ROS level and apoptosis rate in LPS-induced S-AKI mice***

**Figure 4:**
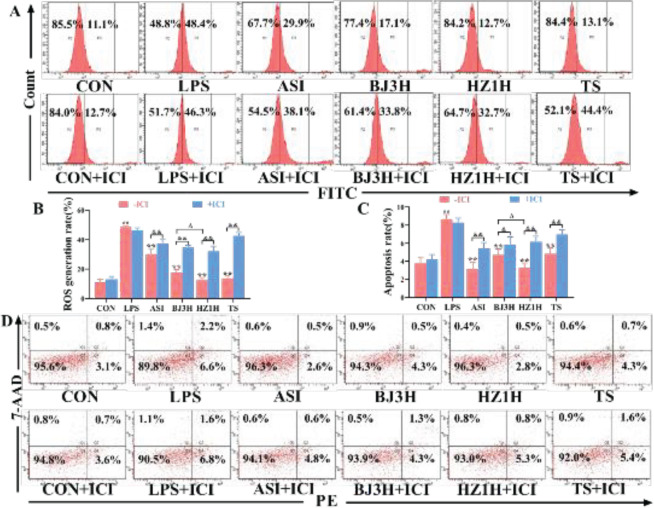
Three types of fresh *Rehmannia glutinosa* reduced the ROS level and apoptosis rate in LPS-induced S-AKI mice. (A‒B) The pictures and quantitative results of the ROS level in mice kidneys. (C‒D) The pictures and quantitative results of the apoptosis rate in mice kidneys. n = 3. CON: normal control group; LPS: model group; ASI: astragalus injection group; BJ3H: Beijing No. 3 fresh *R. glutinosa* water extract group; HZ1H: Huaizhong No. 1 fresh *R. glutinosa* water extract group; TS: Taisheng fresh *R. glutinosa* water extract group. ICI: ICI182,780. ##*P*<0.01 compared with the CON (-ICI) group. **P<0.01 compared with the LPS (-ICI) group. Δ*P*<0.05 compared with the BJ3H (-ICI) group. &*P*<0.05, &&*P*<0.01 for comparison between the groups (± ICI)

The increase of ROS level and apoptosis rate in kidney cells is a prominent and important feature of pathogenesis during S-AKI. The results showed that the levels of ROS and apoptosis rate of primary renal cells in the model group were significantly increased (Figure 4A‒D, *P*<0.01) compared with the control group. After being treated with the drugs, the ROS level and apoptosis rate of primary kidney cells in mice were significantly decreased (*P*<0.01), and the effects of HZ1H were superior to those of BJ3H and TS (*P*<0.05). However, the regulatory effects of each administration group were completely or partially inhibited after adding ICI (*P*<0.05, *P*<0.01). These findings suggested that the three types of *R. glutinosa* may completely or partially reduce the ROS level and apoptosis rate of kidney cells induced by LPS in S-AKI mice through the estrogen receptor pathway.

***Three types of fresh R. glutinosa improved LPS-induced kidney injury in S-AKI mice via the TLR4/NF-κB/NLRP3 signaling pathway***

**Figure 5:**
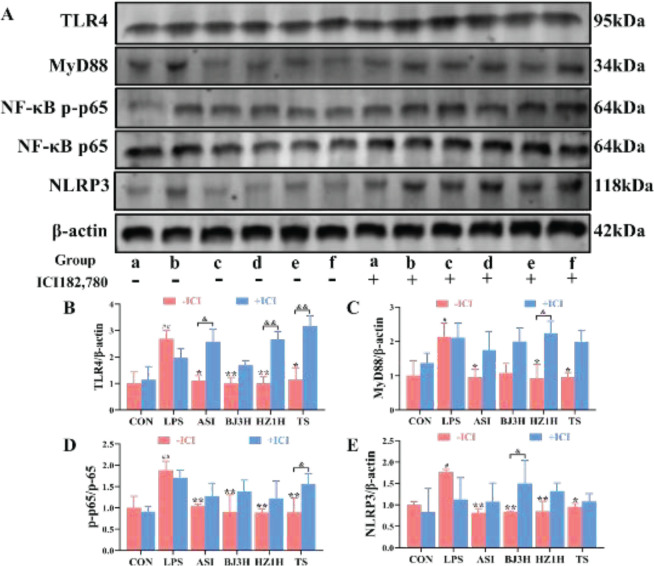
Three types of fresh *Rehmannia glutinosa* improved LPS-induced kidney injury in S-AKI mice via the TLR4/NF-κB/NLRP3 signaling pathway. (A‒E) The expression levels of TLR4, MyD88, NF-κB p-p65, NF-κB p65, and NLRP3 proteins in mice kidneys. n = 3. CON and a: normal control group; LPS and b: model group; ASI and c: astragalus injection group; BJ3H and d: Beijing No. 3 fresh *R. glutinosa* water extract group; HZ1H and e: Huaizhong No. 1 fresh *R. glutinosa* water extract group; TS and f: Taisheng fresh *R. glutinosa* water extract group. ICI: ICI182,780. #*P*<0.05, ##*P*<0.01 compared with the CON (-ICI) group. *P<0.05, **P<0.01 compared with the LPS (-ICI) group. &*P*<0.05, &&*P*<0.01 for comparison between the groups (± ICI)

The expression levels of TLR4, MyD88, and NLRP3 proteins, and the ratio of NF-κB p-p65/NF-κB p65 protein were significantly increased (Figure 5A‒E, *P*<0.05, *P*<0.01) induced by LPS. After treatment with drugs, the expression levels of TLR4, MyD88, NLRP3 proteins, and the ratio of NF-κB p-p65/NF-κB p65 protein in mice kidneys were significantly reduced (*P*<0.05, *P*<0.01), with no significant difference in the regulatory effect of each administration group. However, the effects were completely or partially inhibited by adding ICI (*P*<0.05, *P*<0.01), which indicated that the three types of *R. glutinosa* may completely or partially regulate the TLR4/NF-κB/NLRP3 signaling pathway through the estrogen receptor pathway, thereby improving the LPS-induced inflammation in S-AKI mice and reducing kidney damage.

## Discussion

Sepsis, a syndrome of multiple organ dysfunction caused by a dysregulated response to infection, is characterized by high morbidity and mortality rates (12). Kidneys are one of the most vulnerable organs, and S-AKI is the most common complication of sepsis (13). Studies have shown that astragalus injection has gradually become one of the most commonly used drugs for the treatment of sepsis (14). Some TCM drugs with estrogen-like activity may have an intervening effect on S-AKI through the estrogen receptor pathway (4). However, the overall therapeutic effect of TCM still needs to be improved. *R. glutinosa* is one of the traditional Chinese herbal medicines in China, which is cold and has the effect of clearing heat and cooling blood. It may play a certain role in the treatment of sepsis, which is classified as “heat syndrome” in TCM. At the same time, our previous research found that the water extract of fresh *R. glutinosa* has estrogen-like activity (7). The research team at Henan University of traditional Chinese medicine cultivated new varieties, including HZ1H and TS *R. glutinosa*, from BJ3H *R. glutinosa*. However, the efficacy of these varieties has not been studied. An increasing number of studies have administered an intraperitoneal injection of LPS to establish an *in vivo* model of S-AKI (15-17). Therefore, this experiment further explored the intervention and mechanism of the three types of fresh *R. glutinosa*, namely BJ3H, HZ1H, and TS, on LPS-induced S-AKI mice through the estrogen receptor pathway, and the use of ASI as a positive control. First, three types of fresh *R. glutinosa* water extracts significantly improved the renal pathology and functional damage of the LPS-induced S-AKI mice model. On the whole, HZ1H had better improvement effects than BJ3H and TS. After adding ICI, the effects of the drugs were completely or partially inhibited, suggesting that the three types of *R. glutinosa* may play a protective effect on renal pathology and function in S-AKI mice completely or partially through the estrogen receptor pathway. In addition, it was found that the use of the positive drug ASI also had a similar protective effect.

There are many reasons for the induction of S-AKI by LPS, and its pathological mechanisms mainly include inflammation, immune system damage, excessive production of ROS, and apoptosis (18-21). LPS can induce a systemic inflammatory response, which is the host’s main defense mechanism against invading pathogens. During LPS-induced S-AKI, inflammatory mediators are released in the intravascular compartment and bind to toll-like receptors present on the surface of immune cells such as macrophages and neutrophils, triggering downstream signaling leading to pro-inflammatory synthesis and release of cytokines, including IL-6, TNF-α, IL-1β, and MCP-1 (22). DCs are the main immune cell subset in the kidneys, which can recognize pathogen-associated molecular patterns by recognizing multiple pattern recognition receptors on the surface, such as toll-like receptors and NOD-like receptors, and can modulate AKI-related immune responses (23). The overproduction of ROS is a sign of inflammation, which can directly damage cells and induce increased apoptosis (21). In this study, it was found that the three types of fresh *R. glutinosa* water extracts significantly improved the inflammation, as well as the ROS level and apoptosis rate in LPS-induced S-AKI mice, and regulated the levels of immune cells in the mice kidneys. Moreover, ICI completely or partially inhibited the effects of drugs, which suggested that the three types of fresh *R. glutinosa* may improve inflammation in LPS-induced S-AKI mice completely or partially through the estrogen receptor pathway, thereby regulating the levels of renal immune cells, ROS, and apoptosis, and protecting the pathological process of S-AKI.

To further explore the effects of three types of fresh *R. glutinosa* on the potential anti-inflammatory mechanisms of LPS-induced S-AKI mice model through the estrogen receptor pathway, the expression of TLR4/NF-κB/NLRP3 signaling pathway-related proteins in the mice kidneys were detected by Western blot. LPS can bind to TLR4 and activate MyD88 for signaling, leading to the activation of NF-κB, which in turn activates NLRP3 and promotes the release of inflammatory factors (2). NF-κB is an important transcription factor in the regulation of inflammation and immunity. Inhibiting NF-κB activation during the occurrence of S-AKI can improve AKI (24). The NLRP3 inflammasome is a cytoplasmic macromolecule complex that coordinates the early inflammatory response of the innate immune system. Studies have shown that activated NLRP3 inflammasome can activate the maturation and release of multiple pro-inflammatory cytokines, while inhibition of NLRP3 inflammasome can alleviate AKI (25). The experimental results showed that the three types of fresh *R. glutinosa* reduced the protein expression levels of TLR4, MyD88, NF-κB p-p65/NF-κB p65, and NLRP3 proteins in the mice kidneys. However, ICI completely or partially inhibited the improvement of the drugs. It showed that the three types of *R. glutinosa* may affect the TLR4/NF-κB/NLRP3 signaling pathway completely or partially through the estrogen receptor pathway, improve the inflammatory injury in the LPS-induced S-AKI mice model, and thus play a therapeutic role in S-AKI.

## Conclusion

The three types of fresh *R. glutinosa* water extracts could completely or partially improve the renal pathology and functional damage in LPS-induced S-AKI mice through the estrogen receptor pathway, and regulate inflammation in mice through the TLR4/NF-κB/NLRP3 signaling pathway. Moreover, they can regulate the levels of renal immune cells, ROS, and apoptosis to exert a protective effect on S-AKI, which may provide a certain experimental basis for the clinical treatment of S-AKI. However, the basis of the material action of *R. glutinosa* exerting anti-S-AKI is not clear. So we will mainly focus on the material basis for *R. glutinosa* to improve S-AKI in further study.

## Authors’ Contributions

ML, MZ, and XZ contributed to the experimental design and writing of the manuscript. PG, YZ, XY, JJ, QZ, BZ, BC, and RW performed the experiments and analyzed the data. WF supervised the project. All authors read and approved the final manuscript.

## Data Availability Statement

The datasets generated and/or analyzed during the current study are available from the corresponding author upon reasonable request.

## Ethical Approval

All animal experiments were approved by the ethics committee of Henan University of Chinese Medicine and performed under institutional guidelines. The Ethical Committee approved the animal procedure of the Henan University of Chinese Medicine (DWLL2018080003).

## Conflicts of Interest

The authors declare that they have no conflicts of interest.

## References

[1] Zhang F, Lu S, Jin S, Chen K, Li J, Huang B (2018). Lidanpaidu prescription alleviates lipopolysaccharide-induced acute kidney injury by suppressing the NF-kappaB signaling pathway. Biomed Pharmacother.

[2] Lu P, Zhang L, Liu T, Fan JJ, Luo X, Zhu YT (2021). MiR-494-mediated effects on the NF-kappaB signaling pathway regulate lipopolysaccharide-induced acute kidney injury in mice. Immunol Invest.

[3] Plotnikov EY, Pevzner IB, Zorova LD, Chernikov VP, Prusov AN, Kireev (2019). Mitochondrial damage and mitochondria-targeted anti-oxidant protection in lps-induced acute kidney injury. Anti-oxidants (Basel).

[4] Zeng MN, Zhang L, Zhang BB, Li BK, Kan YX, Yang H (2019). Chinese yam extract and adenosine attenuated LPS-induced cardiac dysfunction by inhibiting RAS and apoptosis via the ER-mediated activation of SHC/Ras/Raf1 pathway. Phytomedicine.

[5] Zhao YY, Chen Y, Han DY, Shan ZF, Li M, Feng WS (2019). Evaluation of the estrogenic effects of rehmapicrogenin. Acta Pharmaceutica Sinica.

[6] State Pharmacopoeia Commission (2020). Pharmacopoeia of the People’s Republic of China: One [M]. Beijing: China Pharmaceutical Science and Technology Press,.

[7] Zheng XK, Liu ZY, Jiang Y, Zhang N, Feng ZY, Su CF (2013). Evaluation of the estrogenic effects of Rehmannia glutinosa libosch. Chinese Pharm. J.

[8] Liu YF, Liang D, Luo H, Hao ZY, Wang Y, Zhang CL (2014). Ionone glycosides from the roots of Rehmannia glutinosa. J Asian Nat Prod Res.

[9] Chen X, Cao YG, Zhang YH, Zeng MN, Ren YJ, Liu YL (2021). Two new ionones from the fresh roots of Rehmannia glutinosa. Phytochemistry Lett.

[10] Liu YL, Cao YG, Kan YX, Ren YJ, Wang MN, Fan XL (2022). Renoprotective activity of a new amide and a new hydroxycinnamic acid derivative from the fresh roots of Rehmannia glutinosa. J Asian Nat Prod Res.

[11] Feng WS, Li M, Zheng XK, Zhang N, Song K, Wang JC (2015). Two new ionone glycosides from the roots of Rehmannia glutinosa Libosch. Nat Prod Res.

[12] Yang J, Miao X, Guan Y, Chen C, Chen S, Zhang X (2021). Microbubble functionalization with platelet membrane enables targeting and early detection of sepsis-induced acute kidney injury. Adv Healthc Mater.

[13] Fan C, Ding X, Song Y (2021). A new prediction model for acute kidney injury in patients with sepsis. Ann Palliat Med.

[14] Xie J, Gong YXY, Ding LS, Luo P, Qing LS (2021). Progress in clinical and pharmacological studies of Astragali Radix and its active components against sepsis. Chinese Herb. Med.

[15] Ma Y, Liu J, Liu H, Han X, Sun L, Xu H (2022). Podocyte protection by Angptl3 knockout via inhibiting ROS/GRP78 pathway in LPS-induced acute kidney injury. Int Immunopharmacol.

[16] Kim K, Leem J (2022). Hispidulin ameliorates endotoxin-induced acute kidney injury in mice. Molecules.

[17] Chancharoenthana W, Udompronpitak K, Manochantr Y, Kantagowit P, Kaewkanha P, Issara-Amphorn J (2021). Repurposing of high-dose erythropoietin as a potential drug attenuates sepsis in preconditioning renal injury. Cells.

[18] Vacas E, Bajo AM, Schally AV, Sanchez-Chapado M, Prieto JC, Carmena MJ (2012). Anti-oxidant activity of vasoactive intestinal peptide in HK2 human renal cells. Peptides.

[19] Xiao L, Ge Y, Sun L, Xu X, Xie P, Zhan M (2012). Cordycepin inhibits albumin-induced epithelial-mesenchymal transition of renal tubular epithelial cells by reducing reactive oxygen species production. Free Radic Res.

[20] Xu F, Zhou H, Wu M, Zhang H, Zhang Y, Zhao Q (2020). Fc-Elabela fusion protein attenuates lipopolysaccharide-induced kidney injury in mice. Biosci Rep.

[21] Wang SC, Zeng MN, Li BK, Kan YX, Zhang BB, Zheng XK (2020). Raw and salt-processed Achyranthes bidentata attenuate LPS-induced acute kidney injury by inhibiting ROS and apoptosis via an estrogen-like pathway. Biomed Pharmacother.

[22] Peerapornratana S, Manrique-Caballero CL, Gomez H, Kellum JA (2019). Acute kidney injury from sepsis: Current concepts, epidemiology, pathophysiology, prevention and treatment. Kidney Int.

[23] Namwanje M, Bisunke B, Rousselle TV, Lamanilao GG, Sunder VS, Patterson EC (2021). Rapamycin alternatively modifies mitochondrial dynamics in dendritic cells to reduce kidney ischemic reperfusion injury. Int J Mol Sci.

[24] Xie Z, Wei L, Chen J, Chen Z (2022). Calcium dobesilate alleviates renal dysfunction and inflammation by targeting nuclear factor kappa B (NF-kappaB) signaling in sepsis-associated acute kidney injury. Bioengineered.

[25] Niu X, Yao Q, Li W, Zang L, Li W, Zhao J (2019). Harmine mitigates LPS-induced acute kidney injury through inhibition of the TLR4-NF-kappaB/NLRP3 inflammasome signalling pathway in mice. Eur J Pharmacol.

